# Hairy Cell Leukemia in a Young Male: An Unusual Presentation

**Published:** 2016

**Authors:** Manveen Kaur, Koushik Kar, Varsha Dalal, Fouzia Siraj

**Affiliations:** *National Institute of Pathology, ICMR, New Delhi, India*

**Keywords:** hairy cell, leukemia, young

**Dear Editor-in-Chief**

A 28 yr male was presented in October 2015 at Medicine Outpatient Department, Safdarjung Hospital, New Delhi- India with generalized weakness, fever and cough for preceding 15 days. Clinicoradiologic examination revealed pallor, firm, non-tender splenomegaly measuring 13.7 cm and hepatomegaly (liver span - 16.9 cm). However, no lymphadenopathy was found. Hemogram showed hemoglobin 6.7 gm/dl, platelet 42000/cmm and total leukocyte count 1710/cmm. Peripheral blood examination revealed a differential count of neutrophils 3%, lymphocytes 66% and monocytes 1%. Atypical lymphoid cells constituted 30% of the differential. A clinical diagnosis of leukemia was made and bone marrow aspiration was done which resulted in ‘dry tap’. Bone marrow biopsy was hypercellular and showed diffuse infiltration by atypical lymphoid cells and few admixed normal hematopoietic cells. The lymphoid cells were small to medium sized with abundant clear cytoplasm giving a “fried egg” appearance (Fig. 1a). On immunohistochemistry, these cells were positive for CD20 and negative for CD34 and CD3. Bone marrow reticulin was significantly increased (3+) (Fig. 1b). Flowcytometric analysis of blood by CD45 vs. side scatter gating cells that expressed bright positivity for CD45, moderate positivity for CD19, CD25, CD103, CD11c, CD123, CD20 and dim positivity for Lambda, CD23, CD10 (Fig 1c). Based on the peripheral blood, bone marrow and immunophenotypic findings, a diagnosis of hairy cell leukemia (HCL) was made.

Informed consent was taken from the patient.

 HCL is a rare form of B cell neoplasm, first described by Bouroncle in 1958 (1). It is insidious in onset, has an indolent course and occurs mainly in elderly male patients. This article reports a case of HCL occurring in a 28 yr old male patient. Worldwide, HCL accounts for only 2% of all leukemias, with a male preponderance (male: female ratio is 4:1) (2). HCL is mainly a disease of middle aged and elderly persons with a median age of 50- 55 yr. The study by Bourancle et al. reported the youngest case being at 22 yr of age (3). Chatterjee et al. (4) reported a case of HCL at 32 yr of age, while the youngest patient of a study by Galani et al. (5) was 26 yr old. Extensive search of the literature revealed only a handful of cases of hairy cell leukemia in patients younger than 30 years of age and no case reports in children (4,5).

Our patient presented with characteristic clinical features of classic HCL. Though exceedingly rare below 30 yr of age, possibility of HCL should always be kept in mind in the setting of appropriate clinical presentation in a young patient; otherwise, this important diagnosis may be missed jeopardizing patient management.

**Fig 1a:**
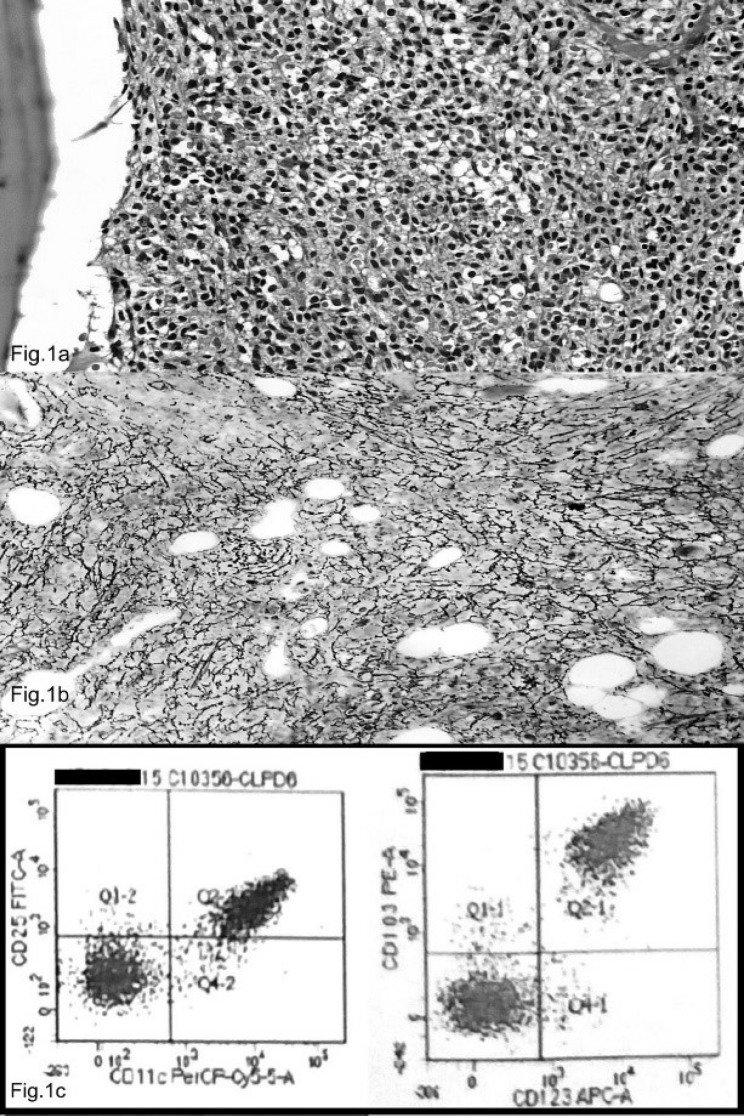
Bone Marrow biopsy showing infiltration by atypical lymphoid cells giving “fried egg” appearance (H & E, x400).Bone Marrow biopsy showing increased reticulin, 3+ (Reticulin stain, x400).Flowcytometric analysis showing increased expression of CD11c, CD25, Cd103 and CD123

## Conflict of Interest:

The authors declare that there is no Conflict of Interests.
